# Impact of proximity of healthier versus less healthy foods on intake: A lab-based experiment

**DOI:** 10.1016/j.appet.2018.10.021

**Published:** 2019-02-01

**Authors:** J.A. Hunter, G.J. Hollands, M. Pilling, T.M. Marteau

**Affiliations:** Behaviour and Health Research Unit, Institute of Public Health, Forvie Site, University of Cambridge School of Clinical Medicine, Box 113 Cambridge Biomedical Campus, Cambridge, CB2 0SR, United Kingdom

**Keywords:** Proximity effect, Position, Healthier food, Less healthy food, Intake

## Abstract

**Background:**

Placing food further away from people decreases likelihood of consumption (“Proximity Effect”). However, it is unclear how proximity affects consumption when both healthier and less healthy foods are available and cognitive resource for self-control is limited.

**Aims:**

To test the hypothesis that when both healthier (raisins) and less healthy (chocolate M&Ms) foods are available, placing less healthy food far, rather than near, increases the likelihood that healthier food is consumed.

**Methods:**

General population participants (*N* = 248) were all put under cognitive load and randomised to one of four groups: 1. Raisins near (20 cm), M&Ms far (70 cm); 2. Both foods near; 3. M&Ms near, raisins far; 4. Both far. Primary outcome: proportions of participants consuming raisins and M&Ms, respectively.

**Results:**

The results did not support the primary hypothesis: when healthier and less healthy foods were both available, placing M&Ms far, rather than near, did not increase likelihood of consuming raisins (*OR* = 1.54, *p* = .432). Regardless of the M&Ms proximity, likelihood of consuming raisins was unaffected by the raisins’ proximity (62.9%(near) vs. 56.5%(far) *OR* = 0.61, *p* = .211). Likelihood of consuming M&Ms non-significantly decreased when they were far and raisins were near, and when both foods were far (*OR* = 2.83, p = .057). Likelihood of consuming M&Ms was affected by M&Ms proximity, being higher when near (68.3%) than far (55.6%), *OR* = 0.39, *p* = .015. Indices of cognitive load impact (higher vs lower) were unrelated to consumption of either food.

**Conclusions:**

Likelihood of consuming a healthier food was unaffected by its proximity and that of a less healthy food. By contrast, likelihood of consuming a less healthy food was influenced by its proximity and possibly by that of a healthier food. These effects need replication in studies designed to detect smaller effect sizes.

**Trial registration:**

This study was registered online with ISRCTN (ISRCTN11740813).

## 1 Introduction

Many people in high-income countries consume diets that undermine their health, including excessive consumption of foods that are high in calories and fat (Monsivais & Drewnowski, 2009), and few fruits and vegetables (Stringhini et al., 2011). Such suboptimal diets significantly contribute to poor health at population level (Newton et al., 2015).

Two key factors that contribute to the high prevalence of unhealthy diets are the presence of food environments characterised by widely available cheap, appealing foods, and the cognitive resources to resist these. Cognitive resources are sets of mental processes comprising intelligence and executive functions (EF). EF includes response inhibition, allowing for self-control of impulsive actions, and support for self-regulation through on-going monitoring of behaviour (Diamond, 2013). Response inhibition varies across individuals both at trait (innate) and state (temporary fluctuations) level. At trait level, the ability to resist reaching for a toy in childhood predicts EF in adolescence, an association considered to be primarily genetic in origin (Friedman, Miyake, Robinson, & Hewitt, 2011). At state level, lower cognitive resource arising from stress (Mani, Mullainathan, Shafir, & Zhao, 2013), or when maintaining items in working memory, which has limited capacity (Engle & Kane, 2004), can negatively impact performance on tasks assessing EF (Mani et al., 2013).

Lower trait cognitive resource is associated with consuming lower quality diets (Cohen, Yates, Duong, & Convit, 2011), overeating (Groppe & Elsner, 2015) and in children is predictive of higher BMI later in childhood (Reinert, Po'e, & Barkin, 2013). Furthermore, when cognitive resource is experimentally lowered, for example through a cognitive load task, food choices shift from healthier to less healthy foods (Shiv & Fedorikhin, 1999; Shiv & Nowlis, 2004; Zimmerman & Shimoga, 2014).

There is preliminary evidence that certain types of dietary interventions, i.e. those that provide information to participants and thus require them to actively reflect on and change behaviour, are more likely to contribute to health disparities between socioeconomic positions (Beauchamp, Backholer, Magliano, & Peeters, 2014; Lorenc, Petticrew, Welch, & Tugwell, 2013; McGill et al., 2015). By contrast, those that have their effects regardless of cognitive resource have the potential to change behaviour across social groups, thereby avoiding their potential to increase disparities by requiring cognitive resource, which is more limited in those living in poverty (Raver, Blair, & Willoughby, 2013). Interventions that alter structural cues in the environment are more likely to operate outside of awareness and therefore not rely, or at least rely less, on cognitive resource (Hollands, Marteau, & Fletcher, 2016; Marteau, Hollands, & Fletcher, 2012) and thus may have the potential to be effective regardless of cognitive resource.

One such intervention involves altering the distance at which food is positioned. Placing a single less healthy food further away reduces the likelihood that it is consumed (Hunter, Hollands, Couturier, & Marteau, 2018; Maas, de Ridder, de Vet, & De Wit, 2012) and how much is consumed (Maas et al., 2012; Musher-Eizenman et al., 2010; Painter, Wansink, & Hieggelke, 2002; Wansink, Painter, & Lee, 2006). This “proximity effect” does not seem to be moderated by cognitive resource (Hunter et al., 2018). Similarly, placing a single healthier food closer increases the likelihood that it is consumed (Musher-Eizenman et al., 2010; Privitera & Creary, 2013). In real-world environments, such as cafeterias, people are more likely to select healthier and less healthy foods when they are placed on the front row in a servery compared to middle or back rows (Meyers & Stunkard, 1980; Rozin et al., 2011) and to purchase less chocolate when it is placed at a more distant till compared to closer, more convenient tills (Meiselman, Hedderley, Staddon, Pierson, & Symonds, 1994). Such evidence of the apparent consistency of the proximity effect across a range of foods suggests that a possible intervention capitalising on these effects could increase intake of healthier food by positioning it closer to people and/or reduce intake of less healthy food by positioning it further away.

There is increasing interest in developing and applying such interventions within real-world settings, involving altering aspects of the small-scale physical environment to influence behaviour, including likelihood to consume and amount of food consumed (Bucher et al., 2016a; Hollands et al., 2013, 2017). However, the current evidence base is limited in a number of important ways making it uncertain whether and how such interventions might be implemented to improve diets at population level. First, most experimental studies of food proximity involve manipulating the distance of a single food (Maas et al., 2012; Musher-Eizenman et al., 2010; Painter et al., 2002; Privitera & Creary, 2013; Wansink et al., 2006), although any intervention capitalising on these effects would likely be implemented in a food environment in which multiple foods varying in healthiness are available. Only a small number of studies have examined the impact on consumption of experimentally varying the proximity of both healthier and less healthy foods (Kroese, Marchiori, & de Ridder, 2015; Meiselman et al., 1994; Meyers & Stunkard, 1980; Privitera & Zuraikat, 2014; Rozin et al., 2011), and these studies have generated inconsistent and incomplete findings. In two studies, moving healthier foods, such as muesli bars and fruits, did not change intake of less healthy foods, such as crisps, chocolate and cakes (Kroese et al., 2015; Meyers & Stunkhard, 1980). In a third study, moving candy to a less convenient location, non-significantly increased consumption of fruit (Meiselman et al., 1994); however, only the distance of the candy was manipulated and thus the study could not determine the effect of moving fruit on candy consumption. In a laboratory study, placing either popcorn or apple slices near to participants increased their consumption regardless of the position of the other food (Privitera & Zuraikat, 2014); however, despite manipulating the distance of both foods, the effect of moving popcorn on consumption of apple slices, and vice versa, was not assessed. In a fifth study, it is unclear whether moving less healthy food affected consumption of healthier foods, and vice versa, since consumption of all foods were assessed together (Rozin et al., 2011).

Based on the proximity effect, we hypothesise that placing a less healthy food further away increases the likelihood that a nearer healthier food is consumed. This primary outcome was chosen because many existing laboratory studies already provide evidence that positioning less healthy food further away reduces its intake, but fewer laboratory studies assess the impact of altering proximity of healthier food.

We designed the current study to provide a robust test of this hypothesis by reducing the risk of bias evident in the existing literature. First, we recruited participants that are representative of general populations and simulated lower cognitive resource by giving participants a cognitive load. Few studies investigate the proximity effect with general population samples including participants with lower cognitive resource. Existing studies assessing the proximity effect often recruit university students or staff (Maas et al., 2012; Meiselman et al., 1994; Painter et al., 2002; Privitera & Creary, 2013; Privitera & Zuraikat, 2014; Wansink et al., 2006), being unrepresentative of the general population in having higher cognitive, material and social resources. Only two studies to date purposefully recruited those likely to have lower cognitive resource and directly investigated the role of cognitive resource on the proximity effect (Hunter et al., 2018). However, these studies only assessed the proximity of a single unhealthy food.

The second way in which we strengthened the design of the current study was by recruiting enough participants to detect predicted effects thereby providing a more reliable and generaliseable estimate of the magnitude of the proximity effect (Bucher et al., 2016b). Existing studies assessing the proximity effect typically have small sample sizes and no power calculations (e.g. Maas et al., 2012; Painter et al., 2002; Privitera & Creary, 2013; Privitera & Zuraikat, 2014; Wansink et al., 2006), and are not pre-registered, limiting the reproducibility of the effects observed in many studies (Button et al., 2013; Munafò et al., 2017).

### 1.1 Study aims

The main aim of the current study is to provide more robust evidence than currently exists for how the proximity effect operates for healthier and less healthy food when both are available.

### 1.2 Hypotheses

#### 1.2.1 Primary hypothesis

When healthier (raisins) and less healthy (M&Ms) foods are both available, placing the M&Ms far (70 cm), rather than near (20 cm), increases the likelihood that the healthier food (raisins) is consumed.

#### 1.2.2 Additional hypotheses

When raisins and M&Ms are both available, it is predicted that:

1. M&Ms are more likely to be consumed when M&Ms are near, regardless of the proximity of raisins.
2. A greater amount of raisins are consumed when raisins are near and M&Ms are far.
3. A greater amount of M&Ms are consumed when M&Ms are near and raisins are far.^1^
4. M&Ms and raisins are each more likely to be consumed when each is near rather than far.

## 2 Methods

### 2.1 Design

The design used a between-subjects experimental design:

1. Both foods were placed near (20 cm)
2. Raisins were placed near (20 cm), M&Ms were placed far (70 cm)
3. M&Ms were placed near (20 cm), raisins were placed far (70 cm)
4. Both foods were placed far (70 cm)

### 2.2 Participants

These comprised 248 members of the general population aged 18 and above, recruited from the Cambridgeshire (UK) area by a research agency.

*Power calculation.* Based on a sample size of 62 per group, a 95% CI for a binomial proportion will be ±13.0% or smaller (84% CI of ±10%). Based on this conservative upper limit for variability, we were able to detect an approximate 20.0% difference between two proportions (Julious, 2004), a difference equivalent to that of the aggregate proportions of participants taking the M&Ms from our previous two studies (Hunter et al., 2018): 69.0 (near group) vs. 48.0 (far group), difference = 21.0%.

*Randomisation.* Participants were allocated to one of four groups using a randomly generated number sequence on Excel by the researcher designed to ensure equal numbers per group, and for the bowls to be randomly positioned either to the right or left of the table.

### 2.3 The intervention

*Foods provided.* The less healthy food was chocolate M&Ms (1000g) and the healthier food was mixed jumbo raisins (a quantity which matched the apparent portion-size of the M&Ms), provided in separate clear glass 1-L bowls. An online study was conducted in which participants rated a selection of healthier and less healthy snacks on their perceived tastiness, appeal and healthiness. Chocolate M&Ms and raisins were chosen as a result of being rated similarly by participants on perceived tastiness and appeal, while differing in perceived healthiness (see supplementary file for a brief report of this online study). A mix of colourful large raisins was used, providing some colour variety in the bowl, as well as a unit size comparable to that of the M&Ms. In a previous study, some participants moved the bowl, thus undermining the intervention (Hunter et al., 2018). In the current study, the bowls were placed on non-slip mats to increase the effort required to move them and thus decrease the chance of participants moving them.

*Distance manipulation.* The distances of 20 cm and 70 cm were chosen in order to replicate previous studies (Hunter et al., 2018; Maas et al., 2012).

**Fig. 1:**
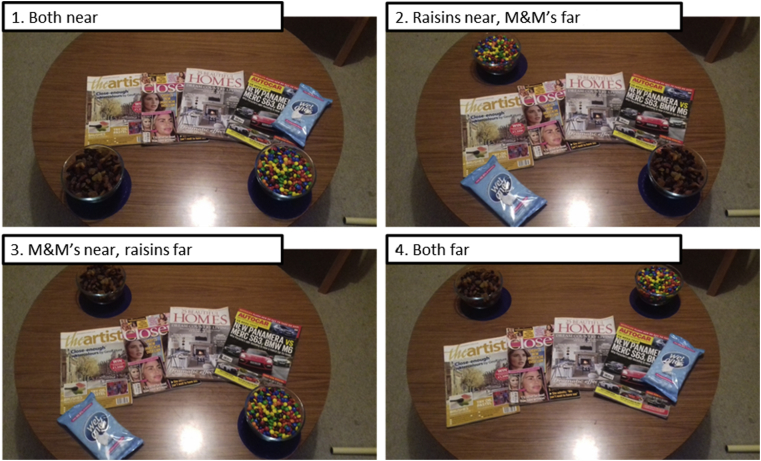
The table layout in each of the four distance groups. *Note* – the images are taken from a standing position where the participant would be seated. In groups 1 and 2, the hand wipes were placed to fill the “unused” space to reduce the novelty of the bowl positions because these groups may have greater overall novelty compared to groups 3 and 4. Fixing the bowls in place was not considered appropriate, as this may have aroused suspicion as to the true nature of the study. Therefore, both bowls were placed on non-slip mats to increase the effort to move the bowl and thus reduce the chance that participants alter the bowls positions.

See Fig. 1 for an image of the table layout in each distance group.

**Cognitive load manipulation.** A string of 7 digits were displayed centrally on a laptop for ten seconds at the start of the session. All participants were given the manipulation, and were required to recall the digits in the correct order later in the study session. This task has been found to impact cognitive resource (Hunter et al., 2018) and dietary behaviour (Shiv & Fedorikhin, 1999; Van Dillen, Papies, & Hofmann, 2013; Zimmerman & Shimoga, 2014).

### 2.4 Outcomes and measures

*Primary outcome.* The proportion (%) of participants who consumed at least some of the raisins was measured as any difference in the raisin bowl's weight.

#### 2.4.1 Additional outcomes

*The proportion (%) of participants who consumed at least some of the M&Ms*. Measured as any differences in the M&Ms bowl's weight.

*Amount of food consumed.* The total amounts of the raisins and M&Ms consumed (grams) was measured from the absolute differences in each bowl weight from before to after the relaxation break.

*Participant food bowl manipulation.* Participants who moved the bowls undermined fidelity of the study protocol. Any bowl manipulation by the participant was recorded and considered in the analysis.

*Education level.* This was measured using a 6-item scale from “no qualifications” to “postgraduate degree”. A dichotomous variable was defined by education level where participants with 5 or more GCSEs/1 A-level were classified as lower education level and participants with 2 or more A-levels and above were classified as higher education level.

*Ethnicity.* This was measured using a 5-item scale including all ethnic backgrounds, A dichotomous variable was defined whereby participants reporting their ethnicity as white were classified as “white” while other ethnicities were classified as “non-white”.

*Participant hunger level.* Hunger was measured using a 7-point rating scale anchored by 1 = “not at all” and 7 = “very”, and operationalised as a dichotomous variable where participants rating 1 and 2 were classified as “not hungry” and those rating 3 or more as experiencing “some hunger” (Forwood, Ahern & Hollands, 2015).

*General liking for each food.* Participants rated on two 100 unit visual analogue scale (VAS) their general liking for chocolate and raisins in response to the statement e.g. “How pleasant would it be to experience a mouthful of chocolate [raisins] now?”, anchored by “not at all” and “extremely” (adapted from Finlayson, King, & Blundell, 2007).

*Cognitive resource.* Behavioural measures of cognitive resource were recorded using the Stroop colour-word interference task (Stroop, 1935) with reaction time (ms) for correct responses to incongruent stimuli (when colour and word meaning did not match) and interference (ms): *mean incongruent latency – [mean congruent latency + mean control trial latency]/2* (Van der Elst, Van Boxtel, Van Breukelen, & Jolles, 2006), as the outcomes.

*Cognitive load.* For additional exploratory analysis, recalling all seven digits correctly was categorised as depicting “higher cognitive resource” and making errors in recall was categorised as depicting “lower cognitive resource”. Due to the lack of standard guidelines for categorisation in similar study designs, this categorisation was made *post-hoc* to maximise statistical power.

See Table 1 for the order of the measures in the study procedure.

**Table 1:** Order of measures and procedures.

Stage of study	Measure/procedure
First Stroop task	Baseline Stroop interference
	Baseline Stroop reaction time
First questionnaire	Gender
	Age
	Ethnicity
	Education level
	Awareness of study aims
	Impulsivity
Cognitive load manipulation	Digit memorisation procedure
Food distance manipulation (“relaxation break”)	Likelihood to consume raisins
	Likelihood to consume M&Ms
	Amount of raisins consumed
	Amount of M&Ms consumed
	Participant bowl manipulation
Final Stroop task	Stroop interference under cognitive load
	Stroop reaction time under cognitive load
Digit recall	Correct recall
Final questionnaire	Awareness of study aims
	Height and weight
	Hunger
	Liking for chocolate and raisins

**Procedure.** Ethics committee approval was sought and obtained from the University of Cambridge Psychology Research Ethics Committee (PRE.2016.088).

Recruitment and testing took place from 30^th^ January until 11th April 2017. Participants were recruited and screened by a research agency and those who were eligible to participate (excluding those with food allergies or intolerance) were invited to the experimental session and allocated an appointment by the agency. All participants gave informed consent before completing the screening questionnaire, and again on entry to the experimental session. Participants were tested individually in sessions lasting up to 1 h, running between 12:00 and 19:30. Participants were recruited into a study of “relaxation and memory”, a cover story in which the two foods could be placed on the table unobtrusively. Participants provided baseline measures of cognitive resource and provided demographic data in a questionnaire. Following this, all participants were instructed to view a 7-digit number on the computer screen for 10 s, and asked to keep this number in mind until they were asked to recall the number later in the session. They were then given a ten-minute “relaxation break” during which the foods were positioned on the table at the pre-specified distances accompanied with a selection of magazines and anti-bacterial hand wipes. The participant was verbally informed that they were free to read the magazines and help themselves to the foods before the researcher left the room for ten minutes. After the break, the participant repeated the measures of cognitive resource to give the impression that the break served as a relaxation intervention. Finally, participants completed ratings of hunger and general liking for each food that they had been provided. Participants were fully debriefed and reimbursed at the end of the session. After the participants left the room, the weight of the bowls was assessed and the food was topped-up, if necessary, after each participant.

## 3 Analysis

Analysis was conducted using SPSS version 22 and R version 3.4.0 (packages: ggplot2 (version 2.2.1: Wickham, 2009), pscl (version 1.4.9: Jackman, 2017) and car (version 2.1–4: Fox & Weisberg, 2011)). All analyses were conducted: 1) including, and 2) excluding participants who moved the bowls.

### 3.1 Primary outcome

*Proportion of participants who consumed the raisins (%).* A logistic regression model was conducted with the probability of participants taking at least some of the raisins as the outcome. The distances of both foods were included separately as factors and together as an interaction term. The model fit was checked using a Hosmer-Lemeshow test and was satisfactory.

### 3.2 Additional outcomes

*Proportion of participants who consumed the M&Ms (%).* The logistic regression model as above was repeated with the probability of participants taking at least some of the M&Ms as the outcome.

*Amount of the raisins consumed (grams)*. This was analysed in two ways: 1) a linear regression (Gamma) was conducted with the amount of the raisins consumed as the outcome. Non-consumers were excluded from this analysis because the continuous intake data including non-consumers were negatively skewed, caused by the preponderance of zero data. The distances of both foods were included separately as factors and together as an interaction term. The fit of the model was checked using a Q-Q plot and a residual deviance test and was satisfactory. 2) Exploratory analysis was conducted including all participants as randomised, including non-consumers, using zero-inflated negative binomial models.

*Amount of the M&Ms consumed (grams).* The analysis as above was repeated with the amount of the M&Ms consumed as the outcome.

*Cognitive load.* As a manipulation check, Mann-Whitney U tests were conducted comparing Stroop scores between those categorised as “lower” and “higher” cognitive resource. This test was most appropriate due to the skewed nature of the Stroop data. Participants taking longer than three standard deviations above the mean time taken (milliseconds (ms)) or less than 200 ms to respond to colour-words were excluded from the analysis (*n* = 3) (based on Strauss, Allen, Jorgensen, & Cramer, 2005). Chi-square analysis was conducted to investigate whether the likelihood that participants consumed each of the foods differed by cognitive load across the proximity conditions.

*Proportion of participants who consumed any food (%).* A logistic regression model was conducted with the probability of participants taking any food, regardless of type of food, as the outcome. Both bowls’ distances were included separately and as an interaction term in the model.

*Total calories consumed (kcal).* A linear regression (Gamma) was conducted with the total amount of the healthier food consumed in participants who consumed at least some of any food. Both bowls’ distances were included separately and as an interaction term in the model.

## 4 Results

*Baseline and demographic characteristics*. A total of 249 (lower education level *n* = 102; higher education level *n* = 147) participants was tested, with an average age of 35.7 years (SD = 12.4). One participant was excluded because they refused to have food products placed in the room, resulting in *n* = 248 participants. Nine participants moved the raisins’ bowl and seven moved the M&Ms bowl. At baseline, participants with lower education level showed slower reaction time (*U* = 4,867, *Z* = - 4.27, *p* < .001) and greater interference (*U* = 5,637, *Z* = −2.88, *p* = .004) on the Stroop task compared to those with higher education level. This difference was also found when participants were under cognitive load (see Table 2).

**Table 2:** Demographic and baseline characteristics of the study sample in each food distance group.

Characteristics	Group				All participants (*N* = 248)
	1.Both near (*n* = 62)	2.Raisins near, M&Ms far (n = 62)	3.M&Ms near, raisins far (*n* = 62)	4.Both far (*n* = 62)	
Age in years (*Mdn, M*(*SD*))	34.0, 35.5(11.7)	34.0, 37.6(13.4)	30.5, 33.3(11.8)	33.0, 36.4(12.6)	33.0, 35.7(12.4)
Gender (%(*n*))					
Male	50.0(31)	50.0(31)	46.8(29)	51.6(32)	49.8(124)
Female	46.8(29)	50.0(31)	53.2(33)	48.4(30)	49.4(123)
Other/not say	3.2(2)	0.0(0)	0.0(0)	0.0(0)	0.8(2)
BMI (*M*(*SD*))	26.2(6.0)	24.7(5.4)	24.9(4.4)	26.4(5.6)	25.6(5.4)
Education (%(*n*))					
<4 GCSEs	22.6(14)	19.4(12)	19.4(12)	22.6(14)	20.9(52)
>5 GCSEs/1A-level	19.4(12)	19.4(12)	22.6(14)	19.4(12)	20.1(50)
>2 A-levels	3.2(2)	8.1(5)	6.5(4)	1.6(1)	4.8(12)
Degree/Diploma	8.1(5)	11.3(7)	6.5(4)	12.9(8)	9.6(24)
Postgraduate	46.8(29)	41.9(26)	45.2(28)	43.5(27)	44.6(111)
Ethnicity (%(*n*))					
White	85.5(53)	80.6(50)	83.9(52)	82.3(51)	82.7(206)
Mixed	6.5(4)	0.0(0)	1.6(1)	1.6(1)	2.4(6)
Asian	6.5(4)	16.1(10)	14.5(9)	14.5(9)	13.3(33)
Black	0.0(0)	0.0(0)	0.0(0)	0.0(0)	0.0(0)
Other/not say	1.6(1)	3.2(2)	0.0(0)	1.6(1)	1.6(4)
Stroop (*Mdn*, *M*(*SD*))					
Reaction time	1399, 1528(599)	1306, 1474(697)	1381, 1451(541)	1587, 1618(526)	1401, 1522(595)
Interference	226, 271(281)	234, 267(261)	175, 246(216)	276, 323(236)	231, 277(249)
Liking choc (*M*(*SD*))	39.6(29.4)	41.6(27.4)	38.0(28.4)	38.8(28.0)	39.4(28.2)
Liking raisins (*M*(*SD*))	34.3(28.8)	28.4(26.3)	29.9(25.8)	31.5(27.0)	30.9(26.9)
Hunger (*M*(*SD*))	2.5(1.5)	2.7(1.7)	3.0(1.6)	2.8(1.6)	2.7(1.6)

**Randomisation checks.** Baseline and demographic characteristics were approximately equal across the four groups and between near and far groups for each food.

**Manipulation checks.** Participants who made errors during digit recall (*n* = 67) showed a slower reaction time (*M* = 1231 (*SD* = 448.9), *U* = 4,621, *p* = .008) and greater interference (*M* = 250.5 (*SD* = 195.8), *U* = 4,986, *p* = .055) in the Stroop task compared to those recalling all digits correctly (*n* = 177, *M* = 1102 (*SD* = 353.9) and *M* = 185.3 (*SD* = 146.3) respectively), indicating that those making errors had lower cognitive resource.

**Summary of covariates.** Liking for chocolate did not predict whether the M&Ms were taken (*U* = 7,738, *p* = .192), but as this was found to affect M&M intake in a previous study (Hunter et al., 2018), it was included as a covariate. Liking for raisins predicted whether the raisins were taken (*U* = 10,224, *p* < .001), and was thus included as a covariate in the analyses. Age showed a small non-significant positive correlation with the amount of raisins consumed, Spearmans' *r*(148) = 0.103, *p* = .107, i.e. the older the participant, the more raisins they consumed, and a negative correlation with amount of the M&Ms consumed, Spearmans’ *r*(153) = −0.242, *p* = .0001, i.e. the younger the participant, the more M&Ms they consumed. As age was also associated with M&Ms consumption in a previous study (Hunter et al., 2018), age was included as a covariate in regression models. As a sensitivity analysis, including education level as a covariate in the models was investigated and did not meaningfully affect the outcomes.

**Primary outcome: proportion of participants consuming at least some raisins.** Likelihood to consume raisins was not related to the distance of the M&Ms (near 61.3% vs. far 58.1%, *OR* = 0.71, *p* = .393) or its own proximity (near 62.9% vs. far 56.5%, *OR*(far) = 0.61, *p* = .211) or the interaction between the foods' proximities (*OR* = 1.54, *p* = .432). Excluding nine (3.6%) participants who moved the raisins’ bowl did not alter the results - see Table 3 for effect sizes. These results were similar regardless of whether covariates were included in the model. There were missing data for liking for raisins from two participants.

**Table 3:** The effect of predictors on the proportion of participants who consumed the raisins.

All participants (*n* = 246)	β (*OR*)	95% Confidence interval		p-value	Effect size, z (*r*)
		Lower	Upper		
Proximity of raisins	−0.49 (0.61)	0.281	1.316	.211	−1.25 (−.079)
Proximity of M&Ms	−0.34 (0.71)	0.328	1.542	.393	−0.86 (−.055)
Interaction	0.43 (1.54)	0.524	4.548	.432	0.79 (.050)
Age	0.01 (1.01)	0.986	1.030	.479	0.71 (.045)
Liking for raisins	0.03 (1.03)	0.016	1.039	<.001	4.58 (.291)
Excl bowl movers (*n* = 237)					
Proximity of raisins	−0.71(0.49)	0.220	1.870	.076	−1.78 (−.116)
Proximity of M&Ms	−0.28 (0.76)	0.344	1.651	.484	−0.70 (−.045)
Interaction	0.55(1.74)	0.578	5.253	.325	0.98 (.064)
Age	0.01 (1.01)	0.987	1.032	.420	0.81 (.053)
Liking for raisins	0.03 (1.03)	1.016	1.040	<.001	4.56 (.296)

*Note:* The reference category for the proximity of each food is “near”.

### 4.1 Additional outcomes

*Proportion of participants consuming at least some M&Ms.* Likelihood to consume M&Ms was significantly related to its own proximity (near 68.3% vs. far 55.6%, *OR* = 0.39, *p* = .015), and this effect was non-significantly affected by the raisins differently in the following scenarios:

a A 61.0% reduction in the probability of taking any M&Ms was found when they were far compared to near, when the raisin bowl was placed near (M&Ms far 48.4% vs. near 71.0%, *OR*(far) = 0.39 [ = 1–0.610], *p* = .015).
b A 31.0% reduction in the probability of taking any M&Ms was found when they were near compared to far, when the raisin bowl was placed far (raisins far 65.6%vs.near 71.0%, OR = 0.69, *p* = .354).
c A 23.2% reduction in taking M&Ms was found when both bowls were far compared to both near (both far 62.9% vs near 71.0%, OR = exp[-0.36-0.94 + 1.04] = 0.77, *p* = .057).

Excluding seven (2.8%) participants who moved the M&Ms bowl did not alter the results – see Table 4 for effect sizes. Excluding covariates from the model, reduced the effect of the interaction between the bowl's proximities (*OR* = 2.32, *p* = .114). There were missing data for liking for chocolate (*n* = 2), and for likelihood to consume M&Ms (*n* = 1).

**Table 4:** The effect of predictors on the proportion of participants who consumed the M&Ms.

All participants (*n* = 245)	β (*OR*)	95% Confidence interval		p-value	Effect size, z (*r*)
		Lower	Upper		
Proximity of raisins	−0.36 (0.69)	0.319	1.497	.354	−0.93 (−.059)
Proximity of M&Ms	−0.94 (0.39)	0.181	0.822	.015	−2.44 (−.156)
Interaction	1.04 (2.83)	0.977	8.337	.057	1.91 (.122)
Age	−0.03 (0.97)	0.950	0.992	.009	−2.63 (−.168)
Liking for chocolate	0.004 (1.00)	0.995	1.014	.375	0.89 (.057)
Excl bowl movers (*n* = 238)					
Proximity of raisins	−0.31 (0.73)	0.327	1.615	.439	−0.78 (−.051)
Proximity of M&Ms	−0.96 (0.38)	0.176	0.817	.014	−2.45 (−.159)
Interaction	0.93 (2.53)	0.854	7.601	.095	1.67 (.108)
Age	−0.03 (0.97)	0.949	0.992	.007	−2.69 (−.174)
Liking for chocolate	0.01 (1.01)	0.996	1.016	.265	1.12 (.073)

*Note:* The reference category for the proximity of each food is “near”.

#### 4.1.1 Amount of the raisins consumed

a. *Participants who consumed at least some raisins.*

The amount of raisins consumed was not related to the proximity of the M&Ms (*OR* = 0.73, *p* = .375). However, it was related to the proximity of the raisins. Placing the raisins far increased the amount of raisins consumed by 69.1% (near *Mdn* = 14.0g (*M* = 18.3, *SD* = 17.6) vs. far *Mdn* = 18.0g (*M* = 26.0, *SD* = 28.9), *OR*(far) = 1.69, *p* = .034. Excluding nine participants who moved the raisins’ bowl reduced the size of this effect (*OR* = 1.43, *p* = .168) – see Table 5 for effect sizes.

b. *All participants as randomised, including non-consumers.*

**Table 5:** The effect of predictors on the amount of the raisins consumed.

All participants (*n* = 147)	β (*OR*)	95% Confidence interval		p-value	Effect size z (*r*)
		Lower	Upper		
Proximity of raisins	0.53 (1.69)	1.046	2.753	.034	2.15 (.177)
Proximity of M&Ms	0.19 (1.21)	0.758	1.943	.424	0.80 (.066)
Interaction	−0.31 (0.73)	0.366	1.460	.375	−0.89 (−.073)
Age	0.01 (1.01)	0.998	1.029	.079	1.77 (.146)
Liking for raisins	0.003 (1.00)	0.996	1.009	.454	0.75 (.062)
Excl bowl movers (*n* = 140)					
Proximity of raisins	0.36(1.43)	0.867	2.411	.168	1.39 (.118)
Proximity of M&Ms	0.20(1.22)	0.764	1.970	.403	0.84 (.071)
Interaction	−0.14(0.87)	0.426	1.778	.709	−0.37 (−.031)
Age	0.02(1.02)	1.001	1.033	.027	2.24 (.189)
Liking for raisins	0.002(1.00)	0.996	1.009	.480	0.71 (.060)

*Note:* The reference category for the proximity of each food is “near”.

Outcomes for the amount consumed are consistent with the model above (see Tables 2.1a and 2.1b zero-inflated models in the supplementary file).

#### 4.1.2 Amount of the M&Ms consumed

a. Participants who consumed at least some M&Ms.

The amount of the M&Ms consumed was unrelated to its own proximity (near *Mdn* = 17.0 (*M* = 24.3, *SD* = 24.0) vs. far *Mdn* = 22.0 (*M* = 27.9, *SD* = 23.0), *OR*(far) = 1.14, *p* = .550) or that of the raisins. These findings did not change when participants who moved the M&Ms bowl were excluded. See Table 6 for effect sizes.

b. *All participants as randomised*, *including non-consumers.*

**Table 6:** The effect of predictors on the amount of the M&Ms consumed.

All participants (*n* = 153)	β (*OR*)	95% Confidence interval		P-value	Effect size z (*r*)
		Lower	Upper		
Proximity of raisins	0.02 (1.02)	0.692	1.497	.933	−0.19 (.015)
Proximity of M&Ms	0.13 (1.14)	0.750	1.741	.550	0.43 (.035)
Interaction	0.16 (1.18)	0.660	2.082	.582	0.40 (.032)
Age	−0.01 (0.99)	0.975	0.998	.026	−2.97 (.240)
Liking for chocolate	0.001 (1.00)	0.996	1.007	.604	0.33 (.027)
Excl bowl movers (*n* = 148)					
Proximity of raisins	0.04 (1.04)	0.699	1.551	.851	−0.06 (.005)
Proximity of M&Ms	0.13 (1.14)	0.747	1.768	.540	0.44 (.036)
Interaction	0.10 (1.11)	0.610	1.995	.739	0.11 (.009)
Age	−0.01 (0.99)	0.975	0.999	.034	−2.83 (.234)
Liking for chocolate	0.002 (1.00)	0.997	1.008	.472	0.57 (.047)

Outcomes are consistent with the model above (see Tables 2.2a and 2.2b zero-inflated models in the supplementary file).

#### 4.1.3 Cognitive resource

Probability of taking raisins (*X*^2^ = 2.15, *p* = .541) and M&Ms (*X*^2^ = 4.31, *p* = .230) did not differ by cognitive resource across the proximity conditions. Probability of taking raisins (*X*^2^ = 0.093, *p* = .761) and M&Ms (*X*^2^ = 2.54, *p* = .111) also did not differ by cognitive resource regardless of proximity condition.

Outcomes for likelihood to consume any food and total calories consumed are available in the supplementary file.

## 5 Discussion

To our knowledge, the current study provides the most direct and robust evidence to date of how the proximity effect operates and interacts with proximity effects of other foods when both healthier (raisins) and less healthy (M&Ms) foods are available. When both foods were available, placing the M&Ms far, rather than near, did not significantly increase the likelihood that the raisins were consumed. In contrast, the likelihood that the M&Ms were consumed was affected by the position of the raisins, such that the likelihood to consume M&Ms was reduced when both M&Ms and raisins were far, or when M&Ms were far and raisins were near, although not to a statistically significant degree, an effect which requires replication. Regardless of where the other food was placed, likelihood to consume raisins was not significantly related to its position, while likelihood to consume the M&Ms was significantly related to its proximity.

We found no evidence to support the primary hypothesis that positioning a less healthy food further away increases the likelihood to consume healthier food. This is inconsistent with the very limited existing evidence which suggests that placing less healthy food far increases the rate of healthier food selection (Meiselman et al., 1994). Though the proximity effect observed for the likelihood of healthier food consumption was in the direction consistent with the existing evidence, i.e. participants were more likely to take the healthier food when it was near compared to far (Meyers & Stunkard, 1980; Privitera & Zuraikat, 2014), this effect was small and statistically non-significant, with the current study not powered to detect an effect of this size. The likelihood of consuming a less healthy food appeared to be affected by the position of the healthier food. Although this effect was statistically non-significant and requires replication, it is inconsistent with the existing evidence from studies using multiple foods where no such effect was observed (Kroese et al., 2015; Meyers & Stunkard, 1980). The observed effects were, however, consistent with the broader evidence that people are less likely to consume, and consume less, of a less healthy food when it is further away compared to near (Hunter et al., 2018; Maas et al., 2012; Meiselman et al., 1994; Meyers & Stunkard, 1980; Musher-Eizenman et al., 2010; Painter et al., 2002; Privitera & Creary, 2013; Wansink et al., 2006).

There are two possible explanations for why the effect of placing raisins near was small and not statistically significant for increasing the likelihood of consuming the raisins. First, the proximity effect may operate differently in a general population sample compared to the student samples used in previous studies where statistically significant proximity effects were found for healthier food (Privitera & Creary, 2013; Privitera & Zuraikat, 2014). Second, cognitive load may affect vulnerability to the proximity effect differently for each food, e.g. people under cognitive load were more likely choose the less healthy of two (equidistant) food choices compared to when not under cognitive load (Shiv & Fedorikhin, 1999).

In terms of the amount of the food consumed, the effect of proximity was inconsistent with existing evidence for both foods i.e. a reverse effect for raisins and no effect for M&Ms. The latter is similar to the lack of effect for the amount of M&Ms consumed in two previous studies (Hunter et al., 2018; see Tables 4.1–4.2b in the supplementary file). There are three possible explanations for the reverse proximity effect for the amount of raisins consumed. First, imbalances in randomisation for unassessed variables could account for some differences in the amount consumed between the proximity conditions. Second, there may have been compensatory effects, i.e. participants may have consumed more raisins when it was far to detract from the urge to consume the M&Ms and to satisfy their craving. Finally, participants may have taken larger handfuls of raisins when the bowl was far due to the greater effort of leaning to obtain it. This suggests that though the proximity effect reduces the likelihood that a food is consumed, it may not consistently affect the amount of the food that is consumed once it is consumed.

A key strength of this study is that it addresses important gaps in the existing literature using a robust study design, extending previous studies manipulating proximity of healthier and less healthy foods (Kroese et al., 2015; Meiselman et al., 1994; Meyers & Stunkard, 1980; Privitera & Zuraikat, 2014; Rozin et al., 2011) often with designs that limited assessment of effects between the foods or which were at high risk of bias. First, by altering the proximity of both healthier and less healthy food, the study directly investigated whether altering proximity of one food affects likelihood to take the other food. Second, this study provides detailed data e.g. odds ratios, interaction-terms and effect sizes for each food at each distance, which should inform power calculations for future studies and aid in reproducibility. Finally, this study recruited a general population sample, including those of lower education level, who had lower manipulated state-level cognitive resource, a sample more representative of those in greatest need of dietary change at population level compared to existing studies. Giving further strength to this study, the statistically significant proximity effect observed in the M&Ms is consistent with previous similar studies using the same or similar foods (Hunter et al., 2018; Maas et al., 2012; Privitera & Creary, 2013).

There were also limitations with this study. First, the study lacked statistical power to detect the smaller than expected proximity effect observed for the raisins and to assess moderation of cognitive load. A larger sample size is required in a future study to address this limitation. Therefore, due to the lack of statistical power, the results of the current cognitive resource analysis are considered as provisional. Second, participants were asked about their liking for chocolate rather than specifically chocolate M&Ms and hunger was assessed at the end of the session to limit any awareness of the true nature of the study earlier in the session, and thus these measures may not have adequately captured the liking and hunger levels which may have initially affected food consumption. Additionally, the size of the proximity effect in situations where healthier and less healthy foods differ in likeability is uncertain. Future studies should assess liking for the specific foods provided. Third, the sample population may not be representative of the UK general population due to a higher proportion of post-graduate participants compared to other levels. Staying in education is associated with cognitive resource (Moffitt et al., 2011); therefore, it is possible that the sample population in this study also had disproportionately higher cognitive resource. Future studies should recruit participants with lower levels of education. Nonetheless, the participants in this study are more representative of the general population in terms of other demographic characteristics, such as age, compared to existing studies assessing the proximity effect, which primarily include students. Finally, this study was conducted under laboratory conditions in which the food-choice task was restrictive, novel and in an artificial setting. Therefore, the observed effects may not be representative of effects in real-world environments in which multiple competing food options are available. Further research should be conducted in real-world settings, such as cafeterias or supermarkets, to assess the generalisability of these outcomes. It is also important that future research investigates whether changes in intake resulting from the proximity effect are sustained in the longer-term (Bucher et al., 2016b).

There is increasing interest in developing and applying interventions which alter aspects of the small-scale physical environment to influence behaviour, including likelihood to consume and amount of food consumed (Bucher et al., 2016a; Hollands et al., 2013, 2017). The effects observed in the current study can inform the development of interventions that involve altering distance of both healthier and less healthy foods relative to each other. For example, such an intervention could place less healthy food further away from till-points in shops or further from consumers in buffet arrangements and would be expected to reduce the likelihood it is consumed. It also appears possible – albeit requiring replication – that the likelihood to consume less healthy food could be further minimised by simultaneously altering the proximity of healthier food options e.g. placing them in nearer and more convenient positions.

## 6 Conclusion

The likelihood to consume healthier, compared with less healthy, food was not significantly affected by its proximity and that of competing less healthy foods. By contrast, the likelihood to consume less healthy food was influenced by its proximity and showed a non-statistically significant decrease when they were far and raisins were near, and when both M&Ms and raisins were far. These differing effects require replication and further testing.

## Author contributions

JAH, TMM and GJH conceived the study. JAH planned and implemented the study and drafted the manuscript with input from TMM and GJH. MP contributed to the drafting of sample size calculation, planned analysis and results section of the manuscript.

## Funding

This research was funded by the Medical Research Council (MRC) and Sackler Prize [MR/K50127X/1], awarded to JAH, The Department of Health Policy Research Program (Policy Research Unit in Behaviour and Health [PR-UN-0409-10109]), and National Institute for Health [NF-SI-0513-10101] awarded to TMM. This report is independent research commissioned and funded by the National Institute for Health Research Policy Research Programme. The views expressed in this publication are those of the author(s) and not necessarily those of the NHS, the National Institute for Health Research, the Department of Health and Social Care or its arm's length bodies, and other Government Department.

## Declaration of interest

The Authors declare they have no conflicts of interest.

## Compliance with ethical standards

All procedures performed in the studies were in accordance with the ethical standards of the institutional research committee and with the 1964 Helsinki declaration and its later amendments. Informed consent was obtained from all individual participants included in the studies.

## Appendix A Supplementary data

Supplementary file Supplementary file

Data Profile Data Profile

## Footnotes

1 In two previous studies (Hunter et al., 2018), the issue of zero-inflated data restricted analysis of amount consumed to only those who consumed at least some of the snacks. However, assessing all participants as randomised, including non-consumers, is the most appropriate way of determining the intervention effect on amount consumed and shows an effect of proximity in existing studies (Maas et al., 2012). Analysis (i.e. including non-consumers) has now also been conducted on data from our two previous studies (Hunter et al., 2018) and has been included in the supplementary file for the current study.
