# Supplementary material for: Impact of proximity of healthier versus less healthy foods on intake: A lab-based experiment
Source: Appetite. 2019 Feb 1;133:147–55. doi: 10.1016/j.appet.2018.10.021 (PMC6335384; doi:10.1016/j.appet.2018.10.021)
Supplement: Supplementary file [file mmc1.docx]

**Supplementary file**

#### Online food rating study

Aim: to select a healthier food option to provide with chocolate M&Ms that matches the M&Ms in perceived tastiness and appeal but differ in perceived healthiness. A search was conducted using Google to select only images that were free to use and share, where the food item filled the whole image and so removed any differences in overall portion size, if they contained small units (e.g. grapes, Maltesers) rather than larger units (e.g. chocolate bars, bananas) and if they did not have wrapping or labels. Eight images were selected in total, with four healthier and four unhealthier foods: chocolate M&Ms, Skittles, Maltesers, Jelly Babies, red grapes, raisins, white grapes and cherry tomatoes.

Participants recruited from the general population (https://www.researchnow.com) completed an online survey (Qualtrics) in which they completed ratings for a selection of healthier and unhealthier food images: 1) “Rate the tastiness of each food item without regard to its healthiness” on a 5-point scale anchored by “Very bad” and “Very good” 2) “Rate the healthiness of each food item without regards to its tastiness” on a 5-point scale anchored by “Very unhealthy” and “Very healthy”, 3) “Rate how appealing each food item looks without regard to its healthiness” on a 5-point scale anchored by “Very unappealing” and “Very appealing”. The mean ratings for each food are presented in *Table 1.1*.

| **Table 1.1:** Food ratings | | | |
| --- | --- | --- | --- |
| **Food** | **Tastiness** | **Healthiness** | **Appeal** |
| Chocolate M&Ms | 3.28 | 1.76 | 3.25 |
| Skittles | 3.12 | 1.78 | 3.13 |
| Maltesers | 3.70 | 2.06 | 3.65 |
| Jelly babies | 3.11 | 1.76 | 3.07 |
| Red grapes | 4.21 | 4.24 | 4.05 |
| Raisins | 3.76 | 4.03 | 3.33 |
| White grapes | 4.15 | 4.24 | 4.03 |
| Cherry tomatoes | 4.02 | 4.41 | 3.79 |

Raisins were selected as the healthier food option since they received equivalent ratings of perceived tastiness and appeal, and differed in perceived healthiness from the M&Ms. A mix of jumbo-sized flame, crimson and golden raisins was selected since it appeared most attractive, juicy and had a variety of colour more similar to the M&Ms than other raisin products.

1. ***Zero-inflated negative binomial model outcomes for the current study***

***Healthier food***

| **Table 2.1a.** Effect of predictors on the amount of the raisins consumed (counts) in zero-inflated negative binomial model | | | | | |
| --- | --- | --- | --- | --- | --- |
| All participants (*n*=248) | β (*OR*) | 95% Confidence interval | | p-value | Effect size z (*r*) |
|  |  | Lower | Upper |  |  |
| Proximity of M&Ms | 0.24 (1.27) | -0.24 | 0.72 | .326 | 0.98 |
| Proximity of raisins | 0.60 (1.82) | 0.10 | 1.09 | .018 | 2.37 |
| Liking for raisins | 0.002 (1.00) | -0.01 | 0.01 | .568 | 0.57 |
| Age | 0.01 (1.01) | -0.001 | 0.03 | .074 | 1.79 |
| Interaction | -0.39 (0.68) | -1.10 | 0.32 | .278 | -1.08 |
| Excl bowl movers (*n*=239) |  |  |  |  |  |
|  |  |  |  |  |  |
| Proximity of M&Ms | 0.26 1.30 | -0.23 | 0.74 | .300 | 1.04 |
| Proximity of raisins | 0.43 1.54 | -0.09 | 0.95 | .106 | 1.62 |
| Liking for raisins | 0.002 1.00 | -0.01 | 0.01 | .611 | 0.51 |
| Age | 0.02 1.02 | 0.002 | 0.04 | .028 | 2.20 |
| Interaction | -0.21 0.81 | -0.94 | 0.52 | .567 | -0.57 |
|  |  |  |  |  |  |

| **Table 2.1b.** Effect of predictors on the amount of the raisins consumed (zeros) in zero-inflated negative binomial model | | | | | |
| --- | --- | --- | --- | --- | --- |
| All participants (*n*=248) | β (*OR*) | 95% Confidence interval | | p-value | Effect size z (*r*) |
|  |  | Lower | Upper |  |  |
| Proximity of M&Ms | 0.56 (1.75) | -0.41 | 1.53 | .255 | 1.14 |
| Proximity of raisins | 0.79 (2.20) | -0.19 | 1.77 | .112 | 1.59 |
| Liking for raisins | -0.03 (0.97) | -0.05 | -0.02 | .0002 | -3.78 |
| Age | -0.01 (0.99) | -0.03 | 0.02 | .646 | -0.46 |
| Interaction | -0.72 (0.49) | -2.02 | 0.58 | .275 | -1.09 |
| Excl bowl movers (*n*=239) |  |  |  |  |  |
|  |  |  |  |  |  |
| Proximity of M&Ms | 0.52 (1.68) | -0.48 | 1.53 | .304 | 1.03 |
| Proximity of raisins | 1.06 (2.89) | 0.02 | 2.11 | .046 | 2.00 |
| Liking for raisins | -0.04 (0.96) | -0.05 | -0.02 | .0003 | -3.62 |
| Age | -0.01 (0.99) | -0.03 | 0.02 | .612 | -0.51 |
| Interaction | -0.87 (0.42) | -2.23 | 0.49 | .209 | -1.26 |
|  |  |  |  |  |  |

*Note:* When including all participants, Theta = 0.92, and when excluding bowl movers, Theta = 0.90.

***Less healthy food***

| **Table 2.2a.** Effect of predictors on the amount of the M&Ms consumed (counts) in zero-inflated negative binomial model | | | | | |
| --- | --- | --- | --- | --- | --- |
| All participants *(n*=247) | β (*OR*) | 95% Confidence interval | | p-value | Effect size z (*r*) |
|  |  | Lower | Upper |  |  |
| Proximity of M&Ms | 0.13 (1.14) | -0.30 | 0.56 | .549 | 0.60 |
| Proximity of raisins | 0.02 (1.02) | -0.37 | 0.41 | .937 | 0.08 |
| Liking for chocolate | 0.002 (1.00) | -0.004 | 0.01 | .599 | 0.53 |
| Age | -0.01 (0.99) | -0.03 | -0.002 | .020 | -2.33 |
| Interaction | 0.17 (1.19) | -0.41 | 0.75 | .575 | 0.56 |
| Excl bowl movers (*n*=240) |  |  |  |  |  |
|  |  |  |  |  |  |
| Proximity of M&Ms | 0.14 (1.15) | -0.30 | 0.57 | .539 | 0.61 |
| Proximity of raisins | 0.04 (1.04) | -0.37 | 0.44 | .853 | 0.19 |
| Liking for chocolate | 0.002 (1.00) | -0.004 | 0.01 | .467 | 0.73 |
| Age | -0.01 (0.99) | -0.03 | -0.002 | .027 | -2.21 |
| Interaction | 0.10 (1.11) | -0.50 | 0.70 | .734 | 0.34 |
|  |  |  |  |  |  |

| **Table 2.2b.** Effect of predictors on the amount of the M&Ms consumed (zeros) in zero-inflated negative binomial model | | | | | |
| --- | --- | --- | --- | --- | --- |
| All participants (*n*=247) | β (*OR*) | 95% Confidence interval | | p-value | Effect size z (*r*) |
|  |  | Lower | Upper |  |  |
| Proximity of M&Ms | 0.98 (2.67) | 0.20 | 1.77 | .015 | 2.44 |
| Proximity of raisins | 0.33 (1.39) | -0.49 | 1.15 | .430 | 0.79 |
| Liking for chocolate | -0.01 (0.99) | -0.02 | 0.01 | .386 | -0.87 |
| Age | 0.03 (1.03) | 0.01 | 0.05 | .012 | 2.50 |
| Interaction | -1.02 (0.36) | -2.14 | 0.10 | .075 | -1.78 |
| Excl bowl movers (*n*=240) |  |  |  |  |  |
|  |  |  |  |  |  |
| Proximity of M&Ms | 1.01 (2.75) | 0.20 | 1.81 | .014 | 2.45 |
| Proximity of raisins | 0.33 (1.39) | -0.51 | 1.18 | .440 | 0.77 |
| Liking for chocolate | -0.01 (0.99) | -0.02 | 0.01 | .285 | -1.07 |
| Age | 0.03 (1.03) | 0.01 | 0.05 | .011 | 2.56 |
| Interaction | -0.96 (0.38) | -2.10 | 0.18 | .200 | -1.65 |
|  |  |  |  |  |  |

*Note:* When including all participants, Theta = 1.30, and when excluding bowl movers, Theta = 1.26.

1. ***Calories consumed***

*Proportion of participants who consumed any food.* The likelihood of consuming at least some food was significantly related to the proximity of the raisins (*OR* = 0.33, *p* = .028), non-significantly by the M&Ms (*OR* = 0.39, *p* = .059) and by the interaction term (*OR* = 5.42, *p* = .013), i.e. the farther away both foods were placed, the less likely any food was consumed. These outcomes did not change when participants who moved either bowl were excluded.

*Total calories consumed.* When analysing only participants who consume any calories, the total amount of calories consumed was unrelated to the proximity of the raisins (*OR* = 1.27, *p* = .200), the M&Ms (*OR* = 1.00, *p* = .993) and the interaction term (*OR* = 1.01, *p* = .975). These outcomes did not change when participants who moved either bowl were excluded.

1. ***Zero-inflated negative binomial model outcomes for the previous studies in Hunter et al. (2018)***

***Study 1***

| **Table 4.1a.** Effect of predictors on the amount of the M&Ms consumed (counts) in zero-inflated negative binomial model | | | | | |
| --- | --- | --- | --- | --- | --- |
| All participants (*n*=159) | β (*OR*) | 95% Confidence interval | | p-value | Effect size z |
|  |  | Lower | Upper |  |  |
| Proximity | -0.03 (0.97) | -0.48 | 0.42 | .888 | -0.14 |
| Education level | 0.19 (1.21) | -0.23 | 0.62 | .368 | 0.90 |
| Ethnicity | -0.11 (0.90) | -0.69 | 0.47 | .708 | -0.37 |
| Hunger | 0.25 (1.28) | -0.20 | 0.69 | .274 | 1.09 |
| Excl bowl movers (*n*=122) |  |  |  |  |  |
|  |  |  |  |  |  |
| Proximity | -0.16 (0.85) | -0.69 | 0.36 | .543 | -0.61 |
| Education level | -0.09 (0.91) | -0.63 | 0.44 | .736 | -0.34 |
| Ethnicity | 0.47 (1.60) | -0.18 | 1.13 | .156 | 1.42 |
| Hunger | -0.30 (0.74) | -0.87 | 0.27 | .302 | -1.03 |
|  |  |  |  |  |  |

| **Table 4.1b.** Effect of predictors on the amount of the M&Ms consumed (zeros) in zero-inflated negative binomial model | | | | | |
| --- | --- | --- | --- | --- | --- |
| All participants (*n*=159) | β (*OR*) | 95% Confidence interval | | p-value | Effect size z |
|  |  | Lower | Upper |  |  |
| Proximity | 0.40 (1.49) | -0.31 | 1.11 | .269 | 1.11 |
| Education level | 0.32 (1.38) | -0.39 | 1.04 | .372 | 0.89 |
| Ethnicity | -0.77 (0.46) | -1.90 | 0.36 | .181 | -1.34 |
| Hunger | -0.24 (0.79) | -0.96 | 0.48 | .512 | -0.66 |
| Excl bowl movers (*n*=122) |  |  |  |  |  |
|  |  |  |  |  |  |
| Proximity | 0.94 (2.56) | 0.12 | 1.76 | .025 | 2.24 |
| Education level | 0.41 (1.51) | -0.42 | 1.23 | .330 | 0.97 |
| Ethnicity | -0.98 (0.38) | -2.25 | 0.28 | .128 | -1.52 |
| Hunger | 0.04 (1.04) | -0.83 | 0.90 | .934 | 0.08 |
|  |  |  |  |  |  |

*Note:* When including all participants, Theta = 1.02, and when excluding bowl movers, Theta = 1.08.

***Study 2***

| **Table 4.2a.** Effect of predictors on the amount of the M&Ms consumed (counts) in zero-inflated negative binomial model | | | | | |
| --- | --- | --- | --- | --- | --- |
| All participants (*n*=246) | β (*OR*) | 95% Confidence interval | | p-value | Effect size z |
|  |  | Lower | Upper |  |  |
| Proximity | 0.07 (1.07) | -0.26 | 0.40 | .693 | 0.40 |
| Cognitive load | 0.07 (1.07) | -0.15 | 0.29 | .556 | 0.59 |
| Age | -0.01 (0.99) | -0.02 | 0.01 | .355 | -0.93 |
| Hunger | -0.09 (0.91) | 0.39 | 0.20 | .531 | -0.63 |
| Load order | -0.12 (0.89) | -0.40 | 0.19 | .474 | -0.72 |
| Interaction prox X load | -0.18 (0.84) | -0.51 | 0.15 | .277 | -1.09 |
| Excl bowl movers (n=226) |  |  |  |  |  |
|  |  |  |  |  |  |
| Proximity | 0.04(1.04) | -0.31 | 0.39 | .829 | 0.22 |
| Cognitive load | 0.07(1.07) | -0.15 | 0.30 | .529 | 0.63 |
| Age | -0.005(1.00) | -0.02 | 0.01 | .447 | -0.76 |
| Hunger | -0.10(0.90) | -0.40 | 0.20 | .517 | -0.65 |
| Interaction prox X load | -0.18(0.84) | -0.52 | 0.17 | .318 | -1.00 |
|  |  |  |  |  |  |

| **Table 4.2b.** Effect of predictors on the amount of the M&Ms consumed (zeros) in zero-inflated negative binomial model | | | | | |
| --- | --- | --- | --- | --- | --- |
| All participants (*n*=246) | β (*OR*) | 95% Confidence interval | | p-value | Effect size z |
|  |  | Lower | Upper |  |  |
| Proximity | 1.40 (4.06) | -0.03 | 2.83 | .055 | 1.92 |
| Cognitive load | 0.19 (1.11) | -0.71 | 0.92 | .803 | 0.25 |
| Age | 0.07 (1.07) | 0.02 | 0.12 | .012 | 2.51 |
| Hunger | 0.53 (1.70) | -0.69 | 1.75 | .397 | 0.85 |
| Load order | -1.34 (0.26) | -2.64 | -0.04 | .044 | -2.01 |
| Interaction prox X load | 0.22 (1.25) | -0.93 | 1.37 | .706 | 0.38 |
| Excl bowl movers (n=226) |  |  |  |  |  |
|  |  |  |  |  |  |
| Proximity | 2.14(8.50) | 0.41 | 3.86 | .015 | 2.43 |
| Cognitive load | 0.02(1.02) | -0.86 | 0.91 | .958 | 0.05 |
| Age | 0.07(1.07) | 0.01 | 0.13 | .024 | 2.25 |
| Hunger | 0.77(2.16) | -0.56 | 2.10 | .257 | 1.13 |
| Interaction prox X load | 0.42(1.52) | -0.81 | 1.64 | .505 | 0.67 |
|  |  |  |  |  |  |

*Note:* This model has a variance that increases quadratically with the mean. The over dispersion parameter is 2.93 in all participants and 2.87 when excluding bowl movers. Load order is included as a predictor in the model with all participants, since this improved the fit of the model. Load order was not included in the model excluding bowl movers, since not having this predictor improved the fit of the model. Load order showed no effect in the model excluding bowl movers.
